# Polyanionic Carboxyethyl Peptide Nucleic Acids (*ce*-PNAs): Synthesis and DNA Binding

**DOI:** 10.1371/journal.pone.0140468

**Published:** 2015-10-15

**Authors:** Yuliya Kirillova, Nataliya Boyarskaya, Andrey Dezhenkov, Mariya Tankevich, Ivan Prokhorov, Anna Varizhuk, Sergei Eremin, Dmitry Esipov, Igor Smirnov, Galina Pozmogova

**Affiliations:** 1 Department of Biotechnology and Bionanotechnology, Moscow State University of Fine Chemical Technologies, Moscow, Russia; 2 Department of Molecular Biology and Genetics, SRI of Physical-Chemical Medicine, Moscow, Russia; 3 Department of Structure-Functional Analysis of Biopolymers, Engelhardt Institute of Molecular Biology, Moscow, Russia; 4 Department of Bioorganic Chemistry, Biology Faculty, Moscow State University, Moscow, Russia; University of Helsinki, FINLAND

## Abstract

New polyanionic modifications of polyamide nucleic acid mimics were obtained. Thymine decamers were synthesized from respective chiral α- and γ-monomers, and their enantiomeric purity was assessed. Here, we present the decamer synthesis, purification and characterization by MALDI-TOF mass spectrometry and an investigation of the hybridization properties of the decamers. We show that the modified γ-*S*-carboxyethyl-T_10_ PNA forms a stable triplex with polyadenine DNA.

## Introduction

Among the various agents that are capable of antigen- and antisense action [1], artificial biopolymers, particularly peptide nucleic acids (PNAs) [2], occupy a special place. A structural unit of PNAs is the *N*-(2-aminoethyl)-glycine (*aeg*) fragment with a nucleic heterocyclic base attached via the acetyl linker. This structure, on the one hand, allows an effective recognition of complementary sequences by nucleic bases [3] due to “restrained flexibility” [4] and, on the other hand, makes *aeg*-PNAs resistant to protease- and nuclease-mediated degradation due to non-natural bonds [5].

The properties of *aeg*-PNAs are well studied. Upon interaction with ss- nucleic acid (NA) targets (deoxyribooligonucleotides (ODNs) or ribooligonucleotides), they form very stable anti-parallel and parallel duplexes [3], and the melting temperatures of the antiparallel duplexes are 10 to 15°C higher than those of the parallel duplexes. High sequence selectivity has been demonstrated for *aeg*-PNAs bases [3, 6], and this selectivity is markedly higher than that found for natural oligonucleotides and their analogues. The formation of triplexes between dsDNA and *aeg*-PNAs has been described [6–7]. Homopyrimidine *aeg*-PNAs are capable of insertion into dsDNA with ssDNA displacement [7]. Additionally, *aeg*-PNAs are capable of forming G-quadruplexes [8].

PNAs have already been investigated for more than 20 years, and a large number of modifications that increase PNA selectivity and affinity for nucleic acid targets or facilitate intracellular PNA delivery have been described [9–11].

A number of studies have shown that the α-*R*- configuration [12–14], β-*S*- [15] and γ-*S*-configurations [16–23] of the acyclic backbone are beneficial for binding; in the latter case, accessible chiral agents, namely *L*-amino acids, were used as the starting compounds. A recent paper [24] describes promising amino- PNA derivatives (aminomethyl α- and γ-PNAs), that exhibited high target affinity and selectivity. The best results were obtained for γ-*S*-derivatives, whereas α-*R*-derivatives were significantly less efficient, and α-S-derivatives had the lowest efficiency. High target affinity has also been reported for oligomers comprising monomers with substituents at both α- and γ- positions with coordinated configurations of the chiral centres (α-*R*-, γ-*S*) [25]. A number of studies have focused on chiral PNAs with guanidine moieties in the side chains [26]. Good cell membrane permeability has been demonstrated for such PNA derivatives, and the binding efficiency of the γ-*S*-derivatives [22, 27–28] was superior to that of the α-*R*-derivatives [28]. All of the above-mentioned studies on PNA modification have aimed at developing new PNA-based molecular tools for fundamental genome studies and broadening the sphere of the practical applications of PNAs [29].

To solve the problems of self-aggregation and poor solubility, two scientific groups have independently proposed phosphono-PNAs [30–32]. ODN binding affinity and selectivity of phosphono-PNAs was inferior to that of *aeg*-PNAs. However, alternation of phosphono- and *aeg*- monomer units in a PNA oligomer resulted in improved binding efficiency [33]. Further improvements were achieved by the alternation of phosphono monomers and cyclic chiral 1*R*, 4*RS*-hydroxyproline-based monomers [34]. Thus, the incorporation of “stereochemically correct” units generally improved the hybridizing properties of such PNAs. Unlike *aeg*-PNA-synthesis, the synthesis of phosphono-PNAs is more similar to that of oligonucleotides than to that of peptides [35]. The preparation of mixed oligomers with *aeg*-units is therefore rather complex [36].

For various phosphono-PNAs derivatives, efficient delivery into cells upon complexation with lipofectamine has been demonstrated [37]. The same delivery principle has been described for *aeg*-PNAs derivatives with negatively charged peptide tails [38]. It has been shown that oligo-*L-*Asp-based fragments conjugated with *aeg*-PNAs are prone to complexation with lipofectamine, and the resulting complexes were readily transported into cells [39]. A longer the polyasparagine tail is associated with a higher the intracellular delivery efficiency.

A number of studies have focused on the incorporation of chiral monomers based on dicarboxylic amino acids into *aeg*-PNA oligomers. Nielsen et al. [13] showed that the introduction of thymine α-monomers based on *D*-glutamic and *L*-asparaginic acids into the H-GT_x_AGAT_x_CACT_x_-NH_2_ *aeg*-PNA decamer decreased the melting temperatures (T_m_) of complexes with native oligonucleotides compared with those of the unmodified PNA/DNA duplex. It has been found that the negatively charged modified decamers demonstrated increased sensitivity to non-complementary nucleosides (mismatches) in the DNA strand (ΔТ_m_^perfect duplex–mismatch duplex^ = 20°C).

In a recent study conducted by Heemstra et al. [40], γ-*S*-carboxymethyl thymine monomers based on *L*-Asp were incorporated into Nielsen’s H-GT_x_AGAT_x_CACT_x_-NH_2_ *aeg*-PNA decamer [13]. The resulting negatively charged PNA oligomers formed rather stable complexes with NA targets (particularly RNA) at moderate and high salt concentrations. Additionally, *aeg*-PNA with γ-*S*-carboxymethyl thymine monomers exhibited high sensitivity to mismatches in both DNA and RNA complements [41].

In recent years, some novel types of negatively charged PNAs have been reported, the and their charges are represented by *N*-alkyl-carboxyl groups in the achiral backbone [42] and sulfomethyl groups at the γ-positions [43]. γ-*S*-Sulfomethyl PNAs formed PNA_2_DNA triplexes and demonstrated good lipofectamine-mediated penetration into SKBR3 cells in model experiments.

It should be noted that all of the previous papers [13, 40, 42–43] describe PNA oligomers with negatively charged thymine monomers exclusively (with at most 3 residues in a chain). To summarize, the incorporation of negative charges into PNA oligomers is required to ensure PNA solubility and compatibility with standard cationic lipofection systems, whereas for an effective recognition of complementary NA sequences the configuration and the positions of chiral centres in the backbone are important. It has been shown earlier that more chiral monomers with the γ-*S* configuration is associated with a the higher NA binding efficiency [16, 23, 27]. However, no previous studies have investigated γ-chiral polyanionic NA mimics with a γ-*S*-chiral centre and a negative charge in each structural unit of the oligomer.

In the present work, we outline a synthesis of homothymine polyanionic PNAs based on *L*-Glu with chiral centres in the γ-*S* configuration (**1, 2**) and an investigation of their hybridization properties with complementary oligodeoxyriboadenylate d(A)_10_. Additionally, this work presents the first synthesis of a homothymine polyanionic PNA α-oligomer (**3**).

## Results and Discussion

### Synthesis of monomers and estimation of their enantiomeric purity

**Fig 1:**
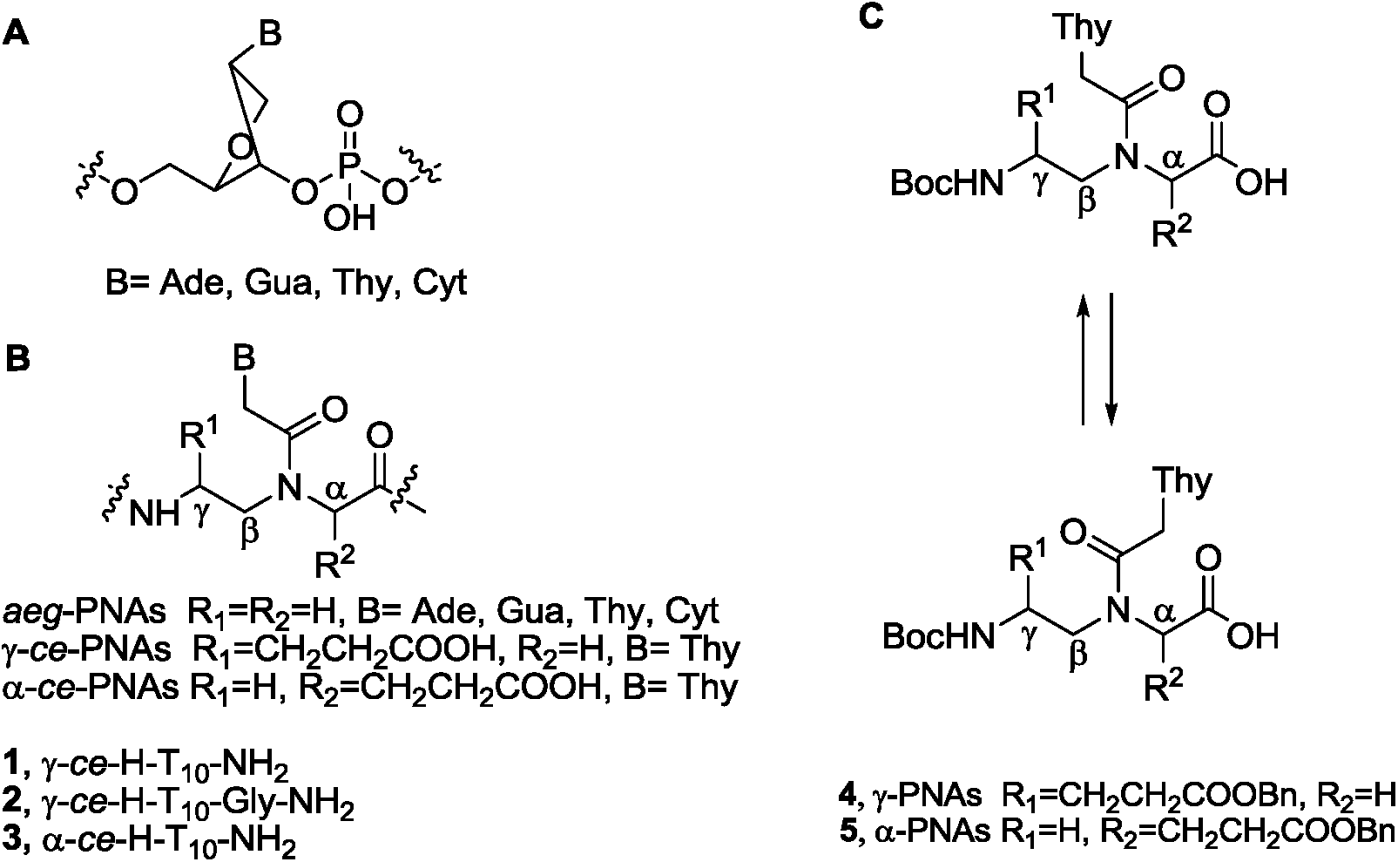
Schematic representation of PNA and DNA fragments. (A) DNA fragment; (B) *aeg*-, γ- and α-*ce*-PNAs; (C) Starting thymine monomers **4** and **5** and their conformers, which coexist in solution due to the reduced rotation along the N^**tert**^-C(O)-bond.

The synthesis of the starting monomers **4** and **5** (Fig 1C) was performed using previously described methods [44–45], in which the key pseudopeptide intermediate was obtained *via* Mitsunobu condensation [46]. In a recent work, we reported the synthesis of monomer **4** *via* thymine alkylation with a bromoacetoamide precursor [47]. An alternative route for the preparation of monomer **5,** in which the pseudopeptide intermediate is obtained by reductive *N*-alkylation, was outlined previously [13]. The syntheses of some other γ-PNA monomers based on dicarboxylic acids and meant for Fmoc-protocol oligomerization (*L*-Asp [40] and *L*-Glu [48]) have also been reported. The preparation of a different type of negatively charged γ-PNA monomers, namely the γ-sulfomethyl PNA monomers, was described previously [43] (the corresponding hydroxymethyl derivatives served as monomer precursors).

Because the stereochemical structure of chiral monomers in PNA oligomers impacts PNA binding with nucleic acid targets [9], we performed enantiomeric purity tests for the starting monomers **4** and **5** using direct and indirect chromatographic methods. The direct approaches for assessing the enantiomeric purity of chiral PNA monomers have been described in the literature [48–49]. Known indirect approaches include preliminary derivatization of *R*- and *S*-PNAs monomers at the *C*-termini with subsequent HPLC-analysis of the resulting diastereomeric mixtures [12–13] and derivatization of γ-chiral monomers at the *N*-termini with Mosher reagents [23–24, 27] with subsequent analysis of the diastereomeric mixtures by ^19^F-NMR spectroscopy. The latter indirect approach was recently used for determining the enantiomeric purity of α- monomers [24], and the purity was shown to be sufficient for subsequent oligomerization. Thus, according to the literature, α- and γ- monomers do not racemize upon *N*-terminal derivatization.

**Fig 2:**
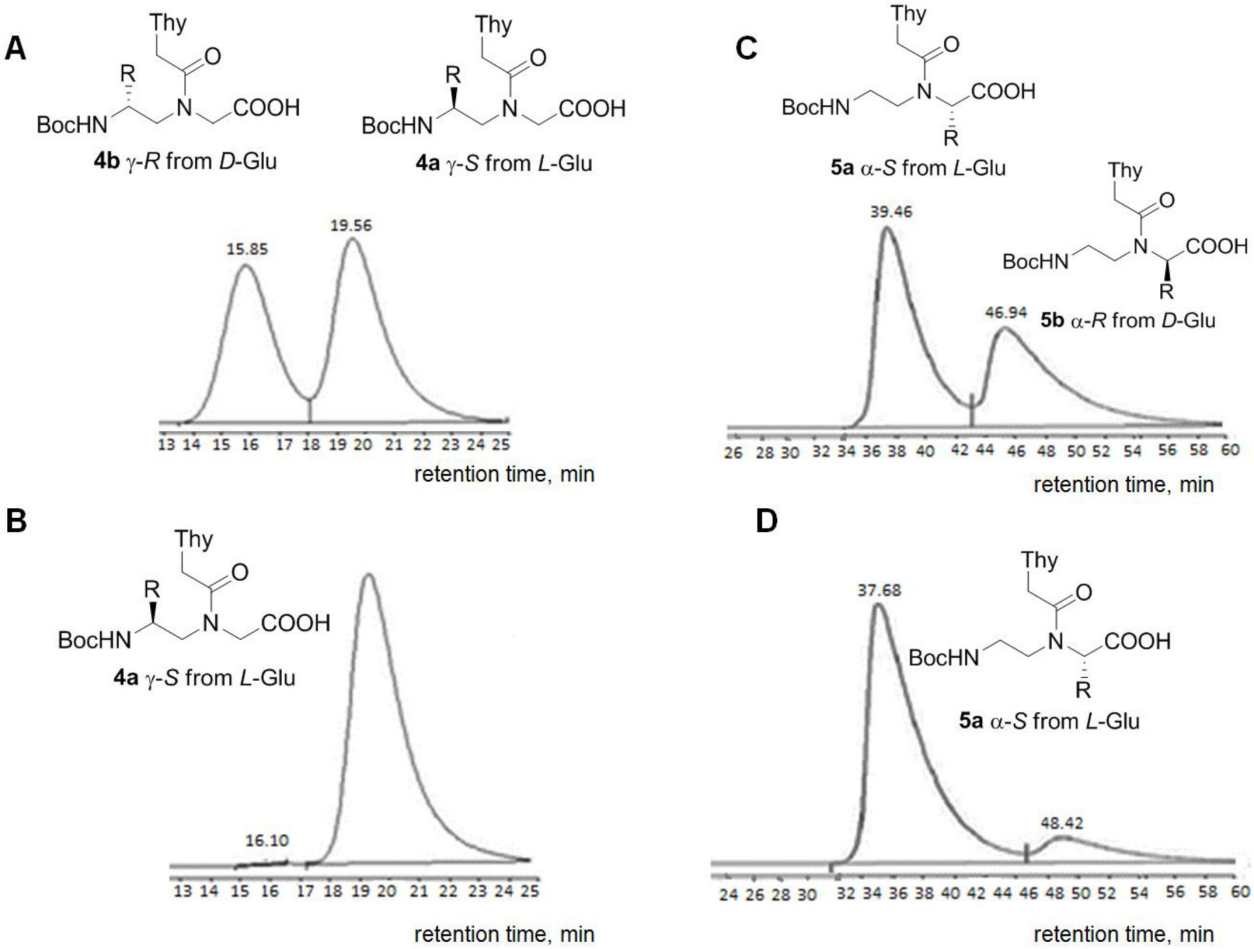
Chromatographic profiles of the PNA monomers (enantiomers). (A) Mixture of **4a** and **4b** (*S*- and *R*- enantiomers of the γ-monomer); (B) *S*-entantiomer of the γ-monomer, **4a** (Conditions: Diasphere column 110-Chirasel-E-PA, 7 μm; elution system: MeOH/CH_3_COOH = 96/4 (v/v); flow rate: 1 mL/min; UV-detection at 254 nm; temperature: 20°C); (C) Mixture of **5a** and **5b** (*R*- and *S*-enantiomers of the α-monomer); (D) *S*-entantiomer of the α-monomer, **5a** (Conditions: Diasphere column 110-Chirasel-E-PA, 7 μm; elution system: MeOH/CH_3_COOH/TEA = 100/0.1/0.1 (v/v/v); flow rate: 0.5 mL/min; UV-detection at 254 nm; temperature: 20°C); R = CH_2_CH_2_COOBn.

The direct approach used in the present work involves the synthesis of two enantiomers (in our case, one from *L*-Glu (γ-*S*-**4a**) and one from *D*-Glu (γ-*R*-**4b**) [44]), the selection of the optimal conditions for their separation on a column with a chiral sorbent and the estimation of the enantiomeric purity in a selected system. For HPLC separation of the **4a/4b** mixture, a hybrid sorbent (silica gel with immobilized eremomycin) was chosen. This chiral selector has been reported to possess good enantio-selectivity toward modified α- and β-amino acids and their derivatives [50–51]. The separation of the *S*- and *R*-monomers, **4a** and **4b**, was relatively efficient under the selected conditions (Fig 2A). The target (γ-*S-*) thymine monomer **4a** was analysed in this system (Fig 2B), and its enantiomeric purity was estimated to be > 99%. The same method was employed to assess the enantiomeric purity of the α-*S*-monomer, **5b** (Fig 2C and 2D). The results of the direct method are shown in Fig 2B and 2D. Peak area calculations indicated that the enantiomeric purity of **5b** was > 99%.

According to the results of the direct tests, both the α- and γ- monomers have sufficient enantiomeric purity and can be used for solid-phase oligomerization without further purification. The significant broadening and asymmetry of the HPLC peaks observed upon separation of the enantiomer mixtures **4a/4b** and **5a/5b** may be due to the existence of each enantiomer in solution as two constrained conformers (Fig 1C). To reliably exclude the presence of substantial enantiomeric impurities, we performed additional indirect tests with monomers **4** and **5**. For these tests, *C*-terminal diastereomers were obtained.

**Fig 3:**
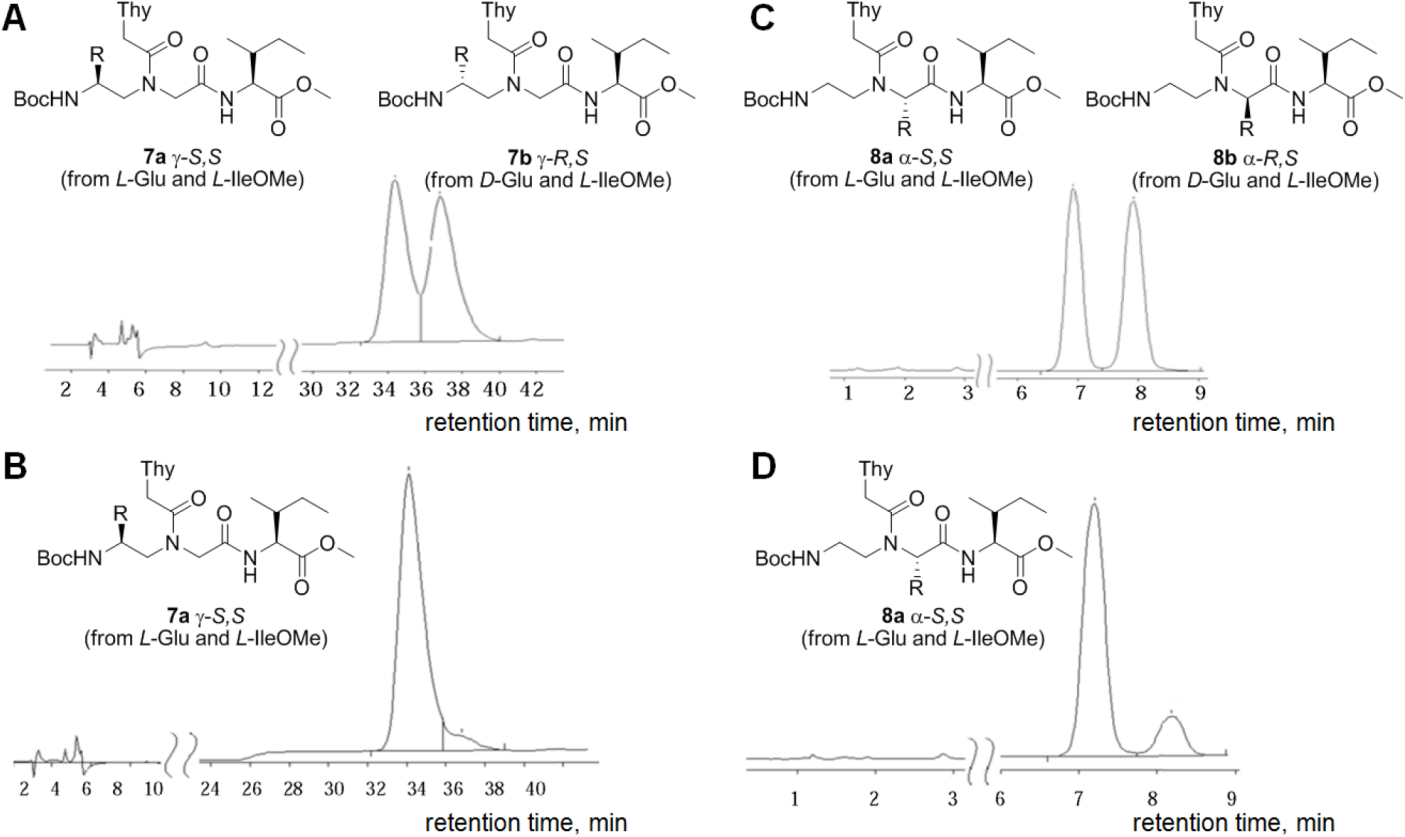
Chromatographic profiles of the PNA monomer derivatives (diastereomers). (A) Mixture of diastereomers **7a** and **7b**; (B) Diastereomer **7a** (Conditions: Luna column, 5 μm, CN 100A, elution system: heptane/isopropanol (88/12), flow rate: 1 mL/min); (C) Mixture of diastereomers **8a** and **8b**; (D) Diastereomer **8a** (Conditions: Separon SGX RP-S C18 column, 5 μm, 4x150 mm, elution system: acetonitrile/water (1/1), flow rate: 1 mL/min); R = -CH_2_CH_2_COOBn.

The enantiomers **4a/4b** and **5a/5b** were derivatized with the asymmetric reagent, *L*-IleOMe **6** (S1 Scheme) in the presence of *N*-ethyl-*N*’-(3-dimethylaminopropyl) carbodiimide (EDC) and 3-hydroxy-1,2,3-benzotriazine-4-on (DHbtOH) to yield the respective diastereomers **7a/7b** and **8a/8b** (Fig 3). The diastereomer structures were confirmed by ^1^H-, and ^13^C-NMR and elemental analysis. Elution systems for reverse-phase HPLC separation of the diastereomer pairs **7a/7b** and **8a/8b** (Fig 3A and 3C, respectively) were selected.

The enantiomeric purities of the target diastereomers **7a** and **8a** (Fig 3B and 3D, respectively) were estimated under the selected conditions. Importantly, no significant peak broadening was observed. Although we were unable to find a system for the complete separation of the diastereomers **7a** and **7b**, the system with heptane/isopropanol (88:12) provided the best separation. The enantiomeric purity of the target *SS*-diastereomer **7a** in this system was approximately 98%. The HPLC analysis of **8 a** revealed that the portions of the *SS*- and *RS*-diastereomers were approximately 90.1 and 9.9%, respectively, which agrees with the results of previous studies [13].

The results of the indirect enantiomeric purity test correlate well with those of the direct test only in the case of the γ-monomer (**4**), but certain racemization may have occurred upon *C*-terminal derivatization of the α-monomer (**5**) to prevent good correlation between the two tests. It can be concluded that the direct tests are more accurate and adequate. It is known that α-PNA monomers have a tendency to racemize upon activation of the *C*-terminal carboxyl group [52]. In this respect, these monomers are not good substrates for oligomerization. In the case of γ-monomers, on the contrary, racemization has never been observed under standard PNA synthesis conditions [53]. Thus, according to our data and the data reported by other groups, γ-monomers do not exhibit a tendency to racemize in indirect tests upon derivatization at both the *N*- and *C*-termini and are stereochemically more stable than α-derivatives. The reproducibility of the results of direct and indirect tests for the γ-monomer **4** confirms this conclusion.

### Solid-phase oligosynthesis

Monomer **4** was used for the synthesis of oligomers **1** and **2.** Monomer **5** was used for the synthesis of oligomer **3**. Solid-phase oligomerization was performed using a modified version of a previously published Boc-protocol for the preparation of *aeg*-PNAs [53]. The resin load of 0.1–0.2 mmol NH_2_-/g, the condensation reagent (HBTU), the Boc-cleavage mixture (TFA with 5% *m*-cresol) and the mixture used for oligomer cleavage from the polymer support were in accordance with the classical protocol. The modifications of the protocol were as follows: capping of the amino groups was performed by treatment with Ac_2_O instead of Rapoport's reagent; [54] 3 (instead of 5) equivalents of the monomer were used at each condensation step; the resin was washed with DMF/DCM instead of Py; and the coupling time was extended from 10–15 minutes to 2 hours.

The thymine oligomer **1** was synthesized on a MBHA-resin using the manual device for solid-phase peptide synthesis (S2 Scheme,). Final deblocking and cleavage of decamer **1** from the polymeric support were performed by the standard «*low-high*» TfOH method, [55] which includes treating the loaded resin with solutions of various trifluoromethanesulfonic acid (TfOH) contents for 1 hour at 0°C (the «*low*» solution (A): TFA−*m*-cresol:DMS:TfOH, 11:2:6:1, v/v/v/v; the «*high*» solution (B): TfOH:TFA:*m*-cresol, 1:8:1, v/v/v). The yield of decamer **1** was 0.7%. The yield of decamer **2** was too low under such conditions; therefore, we introduced several changes into the standard protocol. A glycine spacer between the resin and the growing PNA chain was used in the case of decamer **2**. The deblocking and cleavage of decamer **2** from the support were performed by a previously described method [47]: the resin was pre-cooled to -30°C and then treated with a mixture containing triisopropylsilane (TIS)—TFA:TfOH:TIS (3:1:0.1, v/v/v)—for 1 h at 0°C. Decamer **2** was obtained at a yield of 5.4%.

The α-decamer (**3**) was obtained as a reference oligomer. Different methods were employed for its deblocking and cleavage. Resin treatment with “low” and “high” TfOH solutions for 1 h at 0°C resulted in complete degradation of the target oligomer according to MALDI-MS data. Decreasing the deblocking and cleavage times from 1 hour to 15 and 20 minutes, respectively, allowed us to obtain decamer **3** at a yield of 0.2%, but incomplete cleavage from the support under such conditions is very likely. Resin treatment with a mixture containing triethylsilane (TES)—TFA:TfOH:TES (3:1:0.1, v/v/v)—[56] lead to an insignificant increase in the yield of decamer **3** (0.9%). The maximum yield (2.3%) was obtained upon cleavage with the TFA:TfOH:TIS mixture (3:1:0.1, v/v/v, 1 hr, 0°C) [47].

Oligomers **1, 2** and **3** were purified by reverse-phase HPLC, and their structures were confirmed by MALDI-MS (S1, S2 and S3 Figs).

Because the starting γ-monomer **4** is stereochemically more stable, its oligomerization results in optically pure products, whereas in the case of the α–monomer (**5**) partial racemization during the solid-phase synthesis is possible. To overcome this general problem of α-PNA derivatives, a sub-monomeric approach has been proposed [57–58]; in this approach, carboxymethylated derivatives of the heterocycles are condensed with the pseudopeptide derivative immobilized on the solid phase. Unfortunately, this approach is inapplicable in the case of *L*-Glu-based monomers **4** and **5.** (Monomolecular cyclization of the pseudopeptides would primarily occur instead of the acylation of the secondary amino groups in the respective pseudopeptides with the carboxymethylated heterocycles [59]).

In the early studies on the preparation of α-oligomers, which preceded the emergence of the sub-monomeric approach, the enantiomeric purity of the original monomers (α-*S*- and α-*R*) was ~ 90% according to the results of *C*-terminal derivatization tests [13]. Despite the relatively low purity of the monomers, those studies convincingly demonstrated that the configuration of the chiral centres determines the hybridization properties of α-oligomers to a significant extent.

Thus,γ-derivatives are preferable to α-analogues in terms of the solid-phase synthesis efficiency and the stereochemical stability of the oligomers.

### Hybridization of homothymine *ce*-PNAs with a homoadenine DNA strand

**Fig 4:**
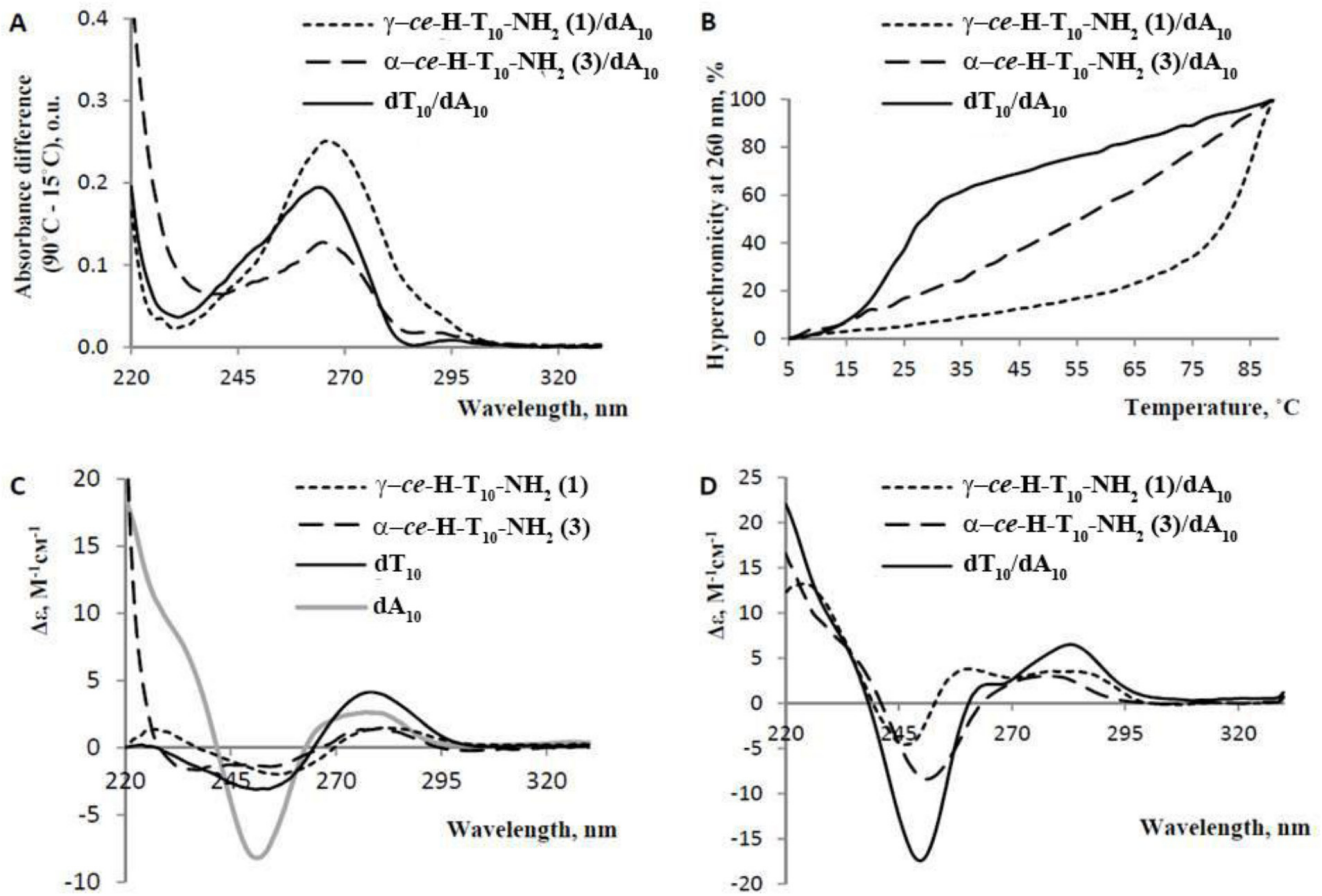
UV-melting and CD spectra of PNA/DNA and DNA/DNA complexes. (A) Thermal difference spectra of the complexes; (B) Thermal denaturation profiles of the complexes; (C) CD spectra of the single-stranded oligonucleotides and PNAs. The molar ellipticity was calculated per 1 nucleotide. (D) CD spectra of the complexes. Conditions: 10 mM Na_2_HPO_4_ (pH 7.4), 140 mM KCl, 5 mM MgCl_2_. The concentration of each PNA or oligonucleotide was 2.5 μM. The CD spectra were measured at 15°C.

The hybridization properties of γ- and α-thymine PNA decamers (**1** and **3**) were studied by physicochemical methods. Natural oligonucleotides that were complementary (dA_10_) and isosequential (T_10_) to α- and γ- PNAs were obtained. Their synthesis, purification and MALDI-MS analysis were performed as described previously [60]. The thermal difference spectra (TDS) from the subtraction of the 15°C spectrum from the 90°C spectrum, CD spectra and UV-melting profiles of the PNA/DNA complexes (Fig 4) were compared with those of the isosequential DNA duplex (dT_10_/dA_10_). The TDS data (Fig 4A) suggested that γ-PNA (**1**) may form either a duplex with increased stability (high TDS amplitude at 260 nm) or a triplex with dA_10_, but the α-PNA (**3**)/dA_10_ complex, if present, was relatively unstable. The TDS curve shapes of both γ-PNA (**1**)/dA_10_ and α-PNA (**3**)/dA_10_ were rather similar to that of the DNA duplex. However the slight bathochromic shift indicates the presence of PNA_2_dA_10_ triplexes [61].

**Fig 5:**
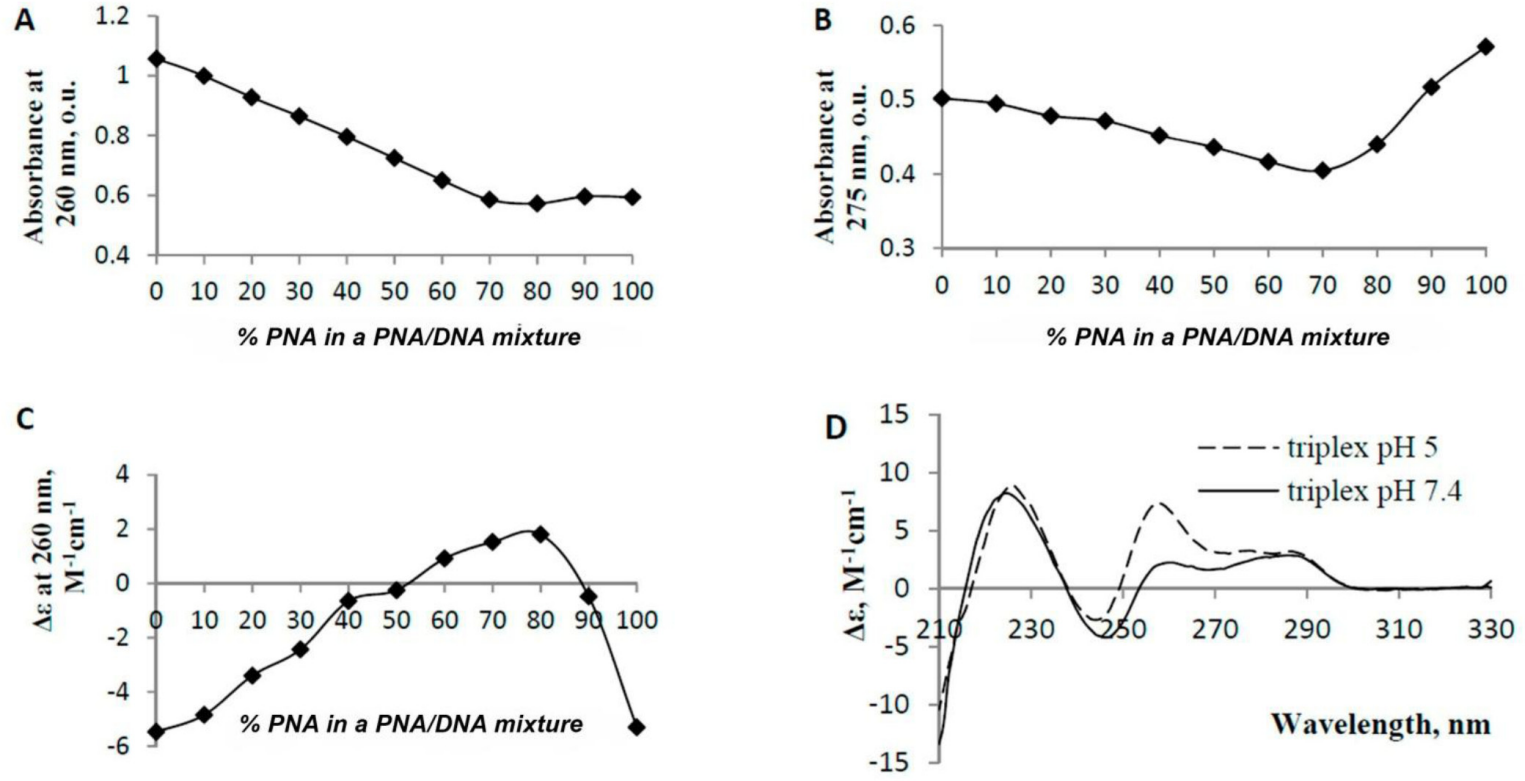
J-Plots and CD spectra of γ-*ce*-H-(T_10_)-Gly-NH_2_ (2)/dA_10_. (A) Absorbance of the γ-*ce*-H-(T_10_)-Gly-OH (**2**)/dA_10_ mixtures with various PNA/DNA ratios at 260 nm; (B) Absorbance of the mixtures at 275 nm; (C) Molar ellipticity at 263 nm. Conditions: 10 mM Na_2_HPO_4_ (pH 7.4), 140 mM KCl, 5 mM MgCl_2_. The summed PNA and DNA concentration was 7 μM at each ratio; (D) CD spectra of the triplex. The molar ellipticity is given per 1 nucleotide in a strand. Conditions: 10 mM Na_2_HPO_4_ (pH 5 or 7.4), 140 mM KCl, 5 mM MgCl_2_. The triplex concentration was 1.8 μM.

The UV-melting data (Fig 4B) also indicate the formation of an extremely stable γ-PNA/dA_10_ complex (T_m_^γ-PNA/dA10^>80°C; T_m_^dT10/dA10^ = 25 ± 1°C) [62]. The melting profile of α-PNA/dA_10_ bears no resemblance to a sigma curve, which may be due to the presence of high-order PNA/DNA structures, their non-cooperative melting or PNA self-aggregation. Decreasing the PNA/DNA or salt concentration up to 5 fold did not change the shape of the α-PNA/dA_10_ melting profile; therefore, it is unlikely that the complex is extremely stable. The CD spectra of the single-stranded PNAs (Fig 4C) are relatively similar to that of dT_10_ in the 244–300 nm wavelength range with the exception of having a decreased amplitude and presenting a hypochromic shift of a negative band. CD spectra of the PNA/dA_10_ complexes (Fig 4C) are substantially different from that of dT_10_/dA_10_, suggesting large differences in geometry or stoichiometry. We prepared a J-plot to clarify the stoichiometry of the γ-*ce*-H-(T_10_)-Gly-NH_2_ (**2**)/dA_10_ complex, as shown in Fig 5A, 5B and 5C. According to the J-Plot results, the PNA:DNA ratio in this extremely stable complex is in the range of 65:35 to 70:30. Therefore, the complex appears to be a PNA:DNA:PNA triplex. This finding agrees with the shape of the CD spectrum of (γ-PNA)_2_dA_10_ (Fig 5D) because the negative band at approximately 210 nm is characteristic of triple helices [63].

The formation of the (γ-PNA)_2_dA_10_ triplex agrees with the data presented in the literature. Analogous PNA_2_DNA triplexes have been reported for homopyrimidine *aeg*-PNA [6] and homothymine chimeric phosphono-PNAs/*aeg*-PNAs [33]. In a recent paper on homopyrimidine decamers containing *aeg*-monomers and thymine monomers with a sulfomethyl substituent at the γ-position, similar triplexes has also been described [43]. The relative orientation of the PNA strands in such complexes has not been unambiguously demonstrated. In DNA homopyrimidine_2_homopurine triplexes [64], the homopyrimidine strands are antiparallel. We hypothesize that this also is likely the case with our (γ-PNA)_2_dA10 triplex, considering its remarkable stability. (A higher stability is characteristic of PNA_2_DNA triplexes that have the Watson–Crick PNA strand in the antiparallel orientation and the Hoogsteen strand in the parallel orientation to the DNA strand [65]).

The sequence-specificity of triplex formation, which is the key aspect in the context of antigen action, was studied. The sensitivity of our γ-PNA decamer to mismatches in a target ODN (S4 Fig) appears to be at least comparable with the sensitivities of homothymine aeg-PNA [6] and homothymine chimeric phosphono-PNA [33], although we were unable to estimate the sensitivity of γ-PNA accurately because of the extreme stability of the (γ-PNA)_2_dA_10_ triplex (even at relatively low ionic strength its T_m_ was > 80°C).

The stability of our (γ-*ce*-T_10_-PNAs)_2_d(A_10_) triplex is close to that described for *aeg*-PNAs [6], which suggests that carboxymethyl groups in a fully charged γ-oligomer do not hamper binding with an ODN target. At the same time, these negatively charged groups ensure PNA compatibility with the conditions required for targeting genomic DNA in culture and potentially *in vivo* (i.e., for PNA-mediated genome editing [66–68] or for gene expression regulation [69–70]). While unmodified *aeg*-PNAs bind DNA most efficiently at low salt concentrations [71], the new polyanionic PNAs work well at both moderate and low ionic strength.

## Conclusions

This study demonstrates the first synthesis of three *L*-Glu-based polyanionic nucleic acid mimics. The steric structures of the new PNA oligomers were predetermined using chiral α- and γ-monomers. The synthesis and purification of the polyanionic PNAs as well as their physicochemical characteristics (particularly, their hybridization affinities toward complementary DNA) were described for α- and γ-thymine decamers.

The results of the hybridization studies suggest that α-*ce*-T_10_-PNAs are not prone to forming stable complexes with DNA and that γ-*ce*-T_10_-PNAs can form triplexes with high thermodynamic stability, similarly to classical *aeg*-PNAs [6]. Our findings indicate the advantages of γ-*ce*-PNAs over α-*ce*-PNAs with respect to complexation with DNA, efficiency of solid-phase synthesis and stereochemical purity of the oligomers. Importantly, our results also suggest that the preorganization of the PNA backbone, which is dependent on the positions and configurations of the chiral centres, may be more significant for molecular recognition than the presence of charged side residues.

In conclusion, it should be noted that further studies of the negatively charged PNAs would require optimization of the solid-phase synthetic protocols. For a more detailed analysis of such compounds, mixed-sequence polyanionic PNAs (i.e., containing different heterocyclic bases) need to be obtained.

## Materials and Methods

The following reagents were used: *L*-H-IleOMe, EDC•HCl, TFA, DMS, TfOH, (Aldrich, USA); HBTU, MBHA-resin (polystyrene resin (4-metylbenzhydryl) amino containing 1% divinylbenzene; 0.5–0.7 mmol/g), acetic anhydride, glacial acetic acid, *m*-cresol, (Acros, USA); DIEA, pyridine (Lancaster, UK); water (Sigma LC-MS, USA); acetonitrile (for spectroscopy, Merck, Germany); DMF, ethanol, methanol, methylene chloride, diethyl ether, ethyl acetate, isopropanol, heptane, P_2_O_5_, EDTA, potassium hydrosulphate, sodium hydrocarbonate, sodium sulphate, ammonium acetate, chloroform-d_1_, sodium chloride, potassium chloride, MgCl_2_•6H_2_O, NaH_2_PO_4_•2H_2_O and Na_2_HPO_4_•12H_2_O.

The following solvents were purified before use: dichloromethane for solid-phase synthesis (washed twice with saturated NaHCO_3_ and twice with water, dried over CaCl_2_ and distilled over P_2_O_5_), ethanol (refluxed with magnesium shavings in the presence of iodine crystals and then distilled), DMF for solid-phase synthesis (maintained over 4-Å molecular sieves for 7 days and distilled over phthalic anhydride *in vacuo*), DIEA (distilled once over ninhydrin and then over KOH), diethyl ether (distilled twice over KOH and, immediately before the reaction, over LiAlH_4_), pyridine (distilled once over ninhydrin and then over KOH), acetonitrile (refluxed over P_2_O_5_ for 2 h and then distilled over P_2_O_5_), triethylamine (distilled over KOH), acetic anhydride (distilled *in vacuo*), trifluoroacetic acid (distilled under atmospheric pressure), trifluoromethanesulfonic (distilled *in vacuo*), *m*-cresol (distilled *in vacuo*), and phenol (distilled *in vacuo*).

Natural oligonucleotides that were complementary (5′-(dA)_10_−3′ and analogues with mismatches) and isosequential (5′-(dT)_10_−3′) to α- and γ- PNAs were synthesized, purified and characterized using MALDI-TOF MS, as described previously [60]. Prior to solid-phase PNA synthesis, the MBHA-resin was prepared as follows: resin was soaked in methylene chloride for 30 minutes and washed with methylene chloride (20 mL/g resin) in a funnel until a clear line of separation appeared. The solvent was then filtered, and the resin was dried under vacuum (oil pump vacuum) and washed on the filter with 5% DIEA in DCM (2x20 mL/g resin) and DCM (2x20 mL/g resin). The solvent was filtered, the resin was dried under vacuum and the number of amino groups on the resin was determined using the quantitative Kaiser test (0.62 mmol-NH_2_/g) [72].

Column chromatography was performed using Merck silica gel 60 (0.040–0.063 mm) with eluents as described below. Thin-layer chromatography (TLC) was performed using Merck silica gel plates 60 F_254_. The TLC spots were visualized under UV light (254 nm), stained with 0.5% ninhydrin solution in ethanol or with a solution of Ce(SO_4_)_2_ and phosphomolybdic acid in 10% aqueous H_2_SO_4_ and heated. The solvents were removed via rotary evaporation under reduced pressure (15 mmHg). The compounds were dried *in vacuo* (2 mmHg).

The NMR spectra were obtained using a Bruker DPX-300 spectrometer. The chemical shifts are given in parts per million (ppm) relative to the signal of the internal standard (tetramethylsilane). The coupling constants are reported in Hertz. The abbreviations used for describing signal multiplicity in the ^1^H-NMR-spectra are the following: s—singlet, d—doublet, dd–double doublet, t–triplet, q—quartet, m—multiplet.

### HPLC conditions for separating enantiomers and diastereomers

The HPLC data were analysed using MicroCal (TM) Origin Version 6.0 (Serial Number G73S4-9478-7000000).

### HPLC analysis and purification of decamers 1, 2 and 3

Analytical HPLC of the PNA oligomers **1**, **2** and **3** (see A, B and C, respectively) was performed as follows: (***A***) A Nucleosil C18 (4.6×250 mm; 5 μm) column was used in an Agilent 1100 chromatography system. Buffer A: 0.1 M AcNH_4_ in water; buffer B: 0.1 M AcNH_4_ in 50% acetonitrile; the gradient of buffer B was 2–12% (linear) over 30 min at a flow rate of 0.85 mL/min, and the eluent was monitored at 260 nm. Temperature: 45°C. (***B***) A Nucleosil C18 (4.6×250 mm; 5 μm) column was used in an Agilent 1100 chromatography system. Buffer A: 0.1 M AcNH_4_ in water; buffer B: 0.1 M AcNH_4_ in 50% acetonitrile; the gradient of buffer B was 5–14% (linear) over 25 min at a flow rate of 0.85 mL/min, and the eluent was monitored at 260 nm. Temperature: 45°C. (***C***) A Nucleosil C18 (4.6×250 mm, 5 μm) column was used in an Agilent 1100 chromatography system. Buffer A: 0.1 M AcNH_4_ in water. Buffer B: 0.1 M AcNH_4_ in 50% acetonitrile. The gradient of buffer B was 4–20% (linear) over 25 min at a flow rate of 0.85 mL/min, and the eluent was monitored at 260 nm. Temperature: 45°C.

The molecular weight of each oligomer was determined through MALDI-TOF MS analysis. The spectra were recorded using a MALDI-TOF MicroFlex mass spectrometer (Bruker, Germany) in the reflectron mode with the detection of positive ions and an acceleration voltage of +20 kV. For all mass spectrometry experiments, recrystallised α-cyano-4-hydroxycinnamic acid (Fluka, USA) was used as the MALDI matrix at a concentration of 25 mM in 50% water/acetonitrile and 0.1% TFA. The matrix and sample were mixed at a 1:1 (v/v) ratio prior to spotting. A total volume of 0.6 μL of the analyte/matrix mixture was deposited for each spot and air dried. A summary spectrum from 200 laser shots was accumulated for every PNA oligomer with the laser intensity was maintained slightly above the ionization threshold.

### Benzyl ester of (*S*) 4-((1*S*, 2*R*)-1-(methoxycarbonyl)-2-methylbutylcarbamoyl)-4-(2-(*N*-*tert*-butyloxycarbonyl)aminoethyl(thymine-1-acetyl))butyric acid (7a)

Monomer **4a** 97 mg (0.178 mmol) was dissolved in 10 mL of DMF, and 44 mg (0.231mmol) of EDC∙HCl was added. After 30 min, 32 mg (0.178 mmol) of HCl∙*L*-Ile-OMe (**6**), 31 mg (0.231 mmol) of DhbtOH and 0.1 mL (0.445 mmol) of TEA were added to the reaction mixture. The mixture was stirred overnight, and then the solvent was evaporated under vacuum. Brine (10 mL) and ethyl acetate were added to the dry residue, and the mixture was extracted with ethyl acetate (2×10 mL). Combined organic fractions were washed with 3 mL of 0.1 M KHSO_4_, NaHCO_3_ (1×5 mL) and brine (1×5 mL), dried over Na_2_SO_4_ and concentrated. The residue was dried under high vacuum. TLC and column chromatography were performed in ethyl acetate. Compound **7a** was obtained at 45% yield (54 mg). *R*_*f*_: 0.46; ^1^H-NMR (CDCl_3_): δ = 9.09, 9.04 (1H, 2s, -NHThy); 7.55–7.28 (5H, m, -Ph); 7.24–7.17, 7.12–7.07 (1H, 2d, *J = 7*.*8 and 8*.*1 Hz*, -C = O-NH(Ile)); 7.00, 6.90 (1H, 2s, C^6^ HThy); 5.80–5.73, 5.72–5.65 (1H, 2t, *J = 6*.*0 and 4*.*1 Hz*, BocNH-); 5.15, 5.11 (2H, 2s, -COOCH_2_Ph); 5.08–4.63 (2H, m; -C = O-CH_2_-Thy); 4.52–4.33 (2H, m; 2αCH-); 3.77, 3.71 (3H, 2s, -OCH_3_); 3.67–3.54 (1H, m, -CH(Ile)); 3.42–3.08 (4H, m, BocNHCH_2_CH_2_); 2.54–2.12 (4H, m, -(γ, β)CH_2_Glu); 1.92, 1.89 (3H, 2s, -CH_3_Thy); 1.43 (9H, s, ^t^Bu-); 1.29–1.22 (2H, q, *J = 3*.*5 Hz*, -CH_2_CH_3_Ile); 0.98–0.84 (6H, m, -2CH_3_(Ile)). ^13^C-NMR (only for one rotamer) (CDCl_3_): δ = 172.9, 172.6, 170.9, 169.4, 164.4, 156.4, 151.1, 141.6, 135.7, 128.6, 128.4, 128.3, 110.5, 110.1, 79.8, 56.9, 52.2, 48.8, 43.7, 39.2, 36.9, 29.7, 28.4, 25.0, 24.3, 15.8, 12.3, 11.6; Anal. Calcd for C_33_H_47_N_5_O_10_: C, 58.83; H, 7.03; N, 10.39. Found: C, 58.88; H, 7.00; N, 9.88.

### Benzyl ester of (*R*) 4-((1*S*, 2*R*)-1-(methoxycarbonyl)-2-methylbutylcarbamoyl)-4-(2-(*N*-*tert*-butyloxycarbonyl)aminoethyl(thymine-1-acetyl))butyric acid (7b)

Compound **7b** was obtained analogously to **7a** from 102 mg (0.187 mmol) of **4b**, 46 mg (0.243 mmol) of EDC∙HCl, 32 mg (0.178 mmol) of HCl∙*L*-Ile-OMe (**6**), 33 mg (0.243 mmol) of DhbtOH and 0.1 mL (0.468 mmol) of TEA. TLC and column chromatography were performed in ethyl acetate. Yield 63 mg (50%). *R*_*f*_: 0.46; ^1^H-NMR (CDCl_3_): δ = 8.94, 8.89 (1H, 2s, -NHThy); 7.44–7.28 (5H, m, -Ph); 7.24–7.18, 7.16–7.09 (1H, 2d, *J = 8*.*2 and 7*.*1 Hz*, -C = O-NH(Ile)); 6.99, 6.90 (1H, 2s, C^6^ HThy); 5.80–5.73, 5.72–5.64 (1H, 2t, *J = 5*.*5 Hz*, BocNH-); 5.15, 5.11 (2H, 2s, -COOCH_2_Ph); 5.08–5.02, 4.95–4.90, 4.88–4.81, 4.78–4.69 (2H, 4d, *J = 14*.*6*, *8*.*5*, *8*.*4 and 16*.*8 Hz*, -C = O-CH_2_-Thy); 4.50–4.33 (2H, m, 2αCH-); 3.77, 3.70 (3H, 2s, -OCH_3_); 3.66–3.53 (1H, m, -CH(Ile)); 3.48–3.05 (4H, m, BocNHCH_2_CH_2_); 2.63–2.50 (1H, m, -CH(Ile)); 2.49–2.02 (4H, m, -(γ, β)CH_2_Glu); 1.89, 1.88 (3H, 2s, -CH_3_Thy); 1.43 (9H, s, ^t^Bu-); 1.29–1.22 (2H, q, *J = 3*.*4 Hz*, -CH_2_CH_3_Ile); 0.94–0.84 (6H, m, -2CH_3_(Ile)). ^13^C-NMR (only for one rotamer) (CDCl_3_): δ = 173.0, 172.5, 170.9, 169.4, 164.4, 156.4, 151.1, 141.1, 135.7, 128.6, 128.4, 128.3, 110.5, 79.8, 66.6, 56.9, 52.2, 48.8, 43.7, 39.2, 36.9, 29.3, 28.9, 25.0, 24.3, 15.8, 12.3, 11.6.

### Benzyl ester of (*S*) 4-(*N*-*tert*-butyloxycarbonyl)amino-5-(carbamoyl-*N*-(1*S*, 2*R*)-1-(methoxycarbonyl)-2-methylbutyl-*N*-(thymine-1-acetyl)amino) pentanoic acid (8a)

Compound **8a** was obtained analogously to **7a** from 100 mg (0.183 mmol) of **5a**, 53 mg (0.277 mmol) of EDC∙HCl, 33 mg (0.181 mmol) of HCl∙*L*-Ile-OMe (**6**), 37 mg (0.274 mmol) of DhbtOH and 0.1 mL (0.458 mmol) of TEA. TLC and column chromatography were performed in ethyl acetate. *R*_*f*_: 0.55, yield: 40 mg (43%); ^1^H-NMR (CDCl_3_): δ = 9.15 (1H, s, -NHThy); 7.41–7.28 (5H, m, Ph); 7.04 (1H, s, C^6^ HThy); 6.89–6.82 (1H, d, *J = 6*.*0 Hz*, -C = O-NH(Ile)); 5.70–5.55 (1H, m, BocNH-); 5.12, 5.10 (2H, 2s, -COOCH_2_Ph); 4.67–4.40 (2H, m, -C = O-CH_2_-Thy); 4.31–4.10 (2H, m, -αCH_2_-(Gly)); 3.99–3.88 (1H, m, -αCH(Ile)); 3.85–3.79 (1H, m, BocNHCHCH_2_-); 3.77, 3.68 (3H, 2s, -OCH_3_); 3.68–3.59 (1H, m, -CH(Ile)); 3.54–3.38 (2H, m, BocNHCHCH_2_-); 2.55–2.40 (2H, m, -(γ)CH_2_Glu); 1.89 (5H, m, -CH_3_Thy, -(β)CH_2_Glu); 1.41, 1.39 (9H, 2s, ^t^Bu-); 1.22–1.10 (2H, m, -CH_2_CH_3_(Ile)); 0.99–0.84 (6H, m, -2CH_3_(Ile)). ^13^C-NMR (only for one rotamer) (CDCl_3_): δ = 173.2, 172.8, 172.4, 168.5, 164.3, 156.0, 151.1, 141.3, 135.8, 128.6, 128.2, 110.6, 79.9, 66.5, 56.7, 52.3, 51.4, 49.5, 48.0, 37.3, 30.8, 28.3, 27.5, 25.2, 15.5, 12.3, 11.5.

### Benzyl ester of (*R*) 4-(*N*-*tert*-butyloxycarbonyl)amino-5-(carbamoyl-*N*-(1*S*, 2*R*)-1-(methoxycarbonyl)-2-methylbutyl-*N*-(thymine-1-acetyl)amino) pentanoic acid (8b)

Compound **8b** was obtained analogously to **7a** from 76 mg (0.139 mmol) of **5b**, 35 mg (0.181 mmol) of EDC∙HCl, 25 mg (0.139 mmol) of HCl∙*L*-Ile-OMe (**6**), 24 mg (0.181 mmol) of DhbtOH and 0.1 mL (0.348 mmol) of TEA. TLC and column chromatography were performed in ethyl acetate. *R*_*f*_: 0.55, yield: 86 mg (70%). ^1^H-NMR (CDCl_3_): δ = 9.15 (1H, s, -NHThy); 7.42–7.28 (5H, m, Ph); 7.06 (1H, s, C^6^ HThy); 6.86–6.79 (1H, d, *J = 6*.*3 Hz*, -C = O-NH(Ile)); 5.51–5.35 (1H, m, BocNH-); 5.12, 5.10 (2H, 2s, -COOCH_2_Ph); 4.61–4.45 (2H, m, -C = O-CH_2_-Thy); 4.28–4.15 (2H, m, -αCH_2_(Gly)); 3.99–3.89 (1H, m, -αCH(Ile)); 3.88–3.78 (1H, m, BocNHCHCH_2_-); 3.76, 3.70 (3H, 2s, -OCH_3_); 3.66–3.55 (1H, m, -CH(Ile)); 3.53–3.37 (2H, m, BocNHCHCH_2_-); 2.54–2.51 (2H, m, -(γ)CH_2_Glu); 1.89 (5H, m, -CH_3_Thy, -(β)CH_2_Glu); 1.40,1.39 (9H, 2s, ^t^Bu-); 1.20–0.82 (8H, m, -CH_2_CH_3_(Ile), -2CH_3_(Ile)). ^13^C-NMR (only for one rotamer) (CDCl_3_): δ = 173.1, 172.9, 172.3, 168.5, 164.3, 156.1, 151.1, 141.3, 135.8, 128.6, 128.2, 110.6, 79.9, 66.5, 56.6, 52.3, 51.2, 49.3, 48.0, 37.3, 30.8, 28.3, 27.5, 25.1, 15.5, 12.3, 11.5.

### Oligomer synthesis

#### Manual solid-phase Boc-protocol synthesis (general procedure)

The negatively charged chiral PNA decamers were prepared on a MBHA resin (0.62 mmol/g) by the Boc-protocol from the C-terminus to N-terminus in the apparatus for manual solid-phase synthesis. All steps were performed at room temperature in a fluid bed. Agitation was performed using inert gas. The procedure included the following steps. 1) Washing the resin twice with DMF/DCM (1:1, v/v) for 2 min, and twice with DMF for 2 min. 2) Coupling with *in situ* neutralization: 0.085 M solution of HBTU (1 eq. per monomer) in DMF was added to a solution of PNA monomer (3 eq.) and DIEA (2 eq. per monomer) in DMF to obtain a final monomer concentration of 0.05 M. Pre-activation was performed for 5 min. The activated solution was transferred to the reactor, and coupling was allowed to proceed for 2 h. 3) Washing with DMF twice for 2 min and twice with DMF/DCM (1:1, v/v) for 2 min. 4) Monitoring coupling using Kaiser tests: if negative, continuing to the next step [72]. Otherwise, repeated coupling was performed under the same conditions. 5) Boc-deprotection with TFA/*m*-cresol (95:5, v/v), 1×10 min, 1×20 min. Steps 1–5 were repeated until the required sequence was obtained.

### Deprotection and cleavage of oligomers from resin

#### Method A—«low-high» TfOH method (general procedure)

The oligomer-resin was dried *in vacuo* (0.1 mm Hg) and cooled on an ice bath for 5 min. Freshly prepared «*low*» TfOH solution (TFA:DMS:*m*-cresol:TfOH, 11:6:2:1) (10 μL per 1 mg resin) was then added at 0°C, and the mixture was allowed to react at ambient temperature for 1 h for PNA deprotection. The solution was filtered, and the resin was washed with TFA (2×4 ml) on the filter. The oligomer was then cleaved from the solid support at 0°C for 1 h using freshly prepared «*high*» TfOH solution (TfOH:TFA:*m*-cresol, 1:8:1) (10 μL per 1 mg resin). The filtrate was separated from the resin by filtration. The resin was washed with TFA (4×4 ml). The filtrate was evaporated and dried for 5 min *in vacuo* (0.1 mm Hg). The product was precipitated with anhydrous diethyl ether and then solubilised in water. The water phase was analysed through HPLC and mass spectrometry.

#### Method B–mild «*low-high*» TfOH method

The only difference from the general procedure (method A) is that the time of resin treatment with «*low*» and «*high*» TfOH solutions was reduced (from 1 h to 15 min for the «*low*» solution and from 1 h to 20 min for the «*high*» solution).

#### Method C—TES method

The oligomer-resin was dried *in vacuo* (0.1 mm Hg). A TFA:TfOH:TES (3:1:0.1) solution (10 μL per 1 mg resin) was then added and allowed to react at 0°C for 1 h. The filtrate was separated from the resin by filtration. The resin was washed with TFA (3×1 ml). The filtrate was evaporated and dried for 5 min *in vacuo* (0.1 mm Hg). The product was precipitated with anhydrous diethyl ether and then solubilised in water. The water phase was analysed through HPLC and mass spectrometry.

#### Method D—TIS method

The oligomer-resin was dried *in vacuo* (0.1 mm Hg) and cooled to -30°C. A TFA:TfOH:TIS (3:1:0.1) solution (10 μL per 1 mg resin) was added and allowed to react at 0°C for 1 h. The filtrate was separated from the resin by filtration. The resin was washed with TFA (3×1 ml). The filtrate was evaporated and dried for 5 min *in vacuo* (0.1 mm Hg). The product was precipitated with anhydrous diethyl ether and then solubilised in water. The water phase was analysed through HPLC and mass spectrometry.

### H-[Thy-(*L*-GluψGly)]_10_-NH_2_ (1)

This negatively charged PNA decamer was assembled analogously to H-[Thy-(Glyψ*L*-Glu)]_10_-NH_2_ (**1**). The synthesis was initiated on 150 mg of MBHA resin with a polymer loading of 0.20 mmol/g. All of the Kaiser tests were negative. The sample was purified by reverse-phase HPLC (***B***) and dried in a SpeedVac: white powder; MS (MALDI-TOF) *m/z*: 3400.0 (calcd. for C_140_H_183_N_41_O_60_: 3399.3). The decamer was cleaved from 57.9 mg of resin by method A. Yield: 257.0 μg (0.7%).

### H-[Thy-(*L*-GluψGly)]_10_-Gly (2)

This negatively charged PNA decamer was assembled analogously to H-[Thy-(*L*-GluψGly)]_10_-NH_2_ (**2**). The synthesis was initiated on 142 mg of MBHA resin with polymer loading of 0.20 mmol/g. The coupling reactions were monitored with Kaiser tests, all of which were negative with the exception of the T-1 and T-7 monomer coupling reactions (double coupling for 2 hr). The sample was purified by reverse-phase HPLC (***C***) and dried in a SpeedVac: white powder; MS (MALDI-TOF) *m/z*: 3457.0 (calcd. for C_142_H_186_N_42_O_61_: 3455.3). The decamer was cleaved from 38.4 mg of resin by method D. Yield: 1.24 mg (5.4%).

### H-[Thy-(Glyψ*L*-Glu)]_10_-NH_2_ (3)

The protected negatively charged PNA decamer was assembled on MBHA resin (150 mg) with a polymer loading of 0.14 mmol/g (determined by quantitative ninhydrin reaction). The coupling reactions were monitored through Kaiser tests, all of which were negative (green-yellow colour with no coloration of the beads) with the exception of the T-2, T-4, T-7 and T-8 monomer coupling reactions (double coupling for 2 h). The sample was purified by reverse-phase HPLC (***A***) and dried in a SpeedVac: white powder; MS (MALDI-TOF) *m/z*: 3400.0 (calcd. for C_140_H_183_N_41_O_60_: 3399.3). The decamer was cleaved from 11 mg of resin by method A (complete degradation), from 9.0 mg of resin by method B with a yield of 7.8 μg (0.2%); from 10.2 mg of resin by method C with a yield of 38.3 μg (0.9%) and from 14.5 mg of resin by method D with a yield of 141.2 μg (2.3%).

### Hybridization studies

The UV-spectra and thermal denaturation profiles of the PNA/DNA complexes and the DNA duplex were recorded with a Jasco UV 750 spectrophotometer (Japan) and a temperature controlled cell in 5 mM sodium phosphate buffer, 140 mM KCl, 5 mM MgCl_2_ (pH 7.46). The concentration of each oligonucleotide or PNA was 5 μM. The samples were denatured at 95°C for 5 min and slowly cooled to 20°C prior to analysis. The absorbance at 260 nm was measured as a function of temperature. The data were recorded every 0.5°C from 5 to 90°C. The melting temperature of the DNA duplex and the mismatched PNA_2_DNA triplexes were defined as the maxima of the first derivatives of absorption from temperature using MicroCal (TM) Origin Version 6.0 (Serial Number G73S4-9478-7000000). Circular dichroism (CD) spectra were obtained with a Jasco J-715 spectropolarimeter at 15°C. The samples were annealed in the same buffer and under the same conditions as used in thermal denaturation studies. The CD values (Δε) are provided per mole of nucleotide.

## Supporting Information

S1 Fig Analytical HPLC profile (A) and MALDI-TOF spectrum (B) of PNA decamer 1. (PDF)

S2 Fig Analytical HPLC profile (A) and MALDI-TOF spectrum (B) of PNA decamer 2. (PDF)

S3 Fig Analytical HPLC profile (A) and MALDI-TOF spectrum (B) of PNA decamer 3. (PDF)

S4 Fig Melting curves of γ-PNA_2_DNA triplexes (fully matched and mismatched). (A) Buffer: 10 mM Na_2_HPO_4_ (pH 7.4), 140 mM KCl, 5 mM MgCl_2_. The concentration of each oligonucleotide or PNA was 2.5 μM. (B) Buffer: 10 mM Na_2_HPO_4_ (pH 7.4), 10 mM KCl. The concentration of each oligonucleotide was 1 μM; PNA concentration was 2 μM. Tm values were defined as the maxima of the first derivatives of absorption from temperature.
(PDF)

S1 Scheme Synthesis of the PNA monomer derivatives (diastereomers). (PDF)

S2 Scheme Synthesis of PNA oligomers. (PDF)
