# Supplementary figures and images for: Polyanionic Carboxyethyl Peptide Nucleic Acids (ce-PNAs): Synthesis and DNA Binding
Source: PLoS One. 2015 Oct 15;10(10):e0140468. doi: 10.1371/journal.pone.0140468 (PMC4607454; doi:10.1371/journal.pone.0140468)

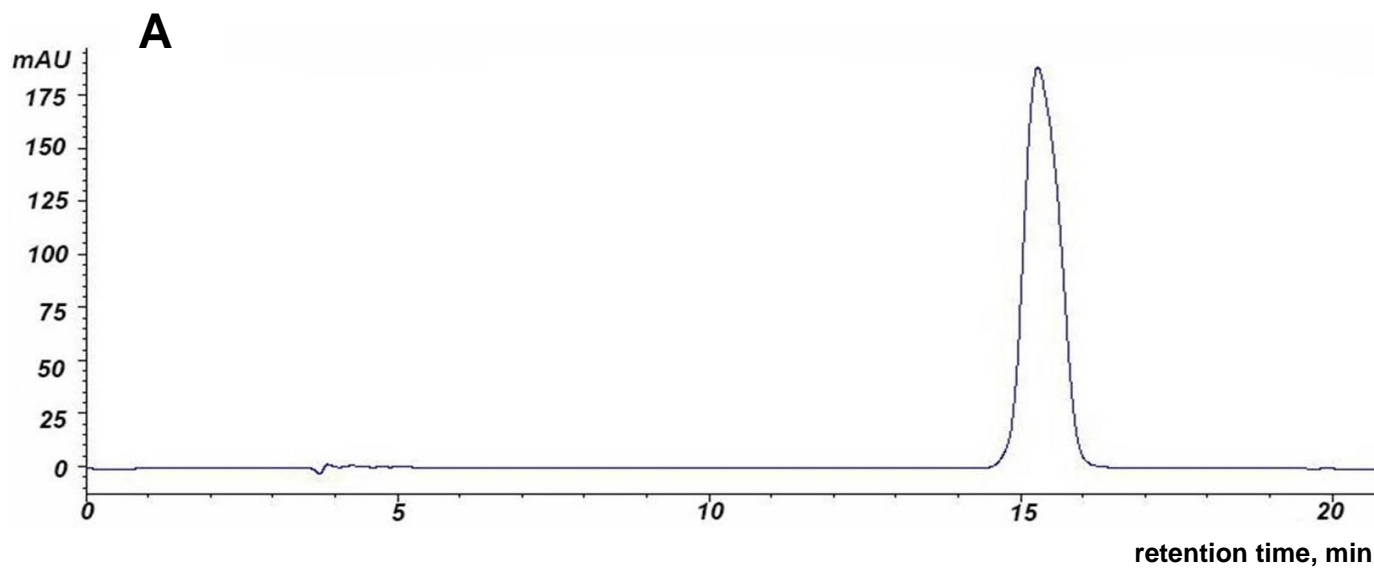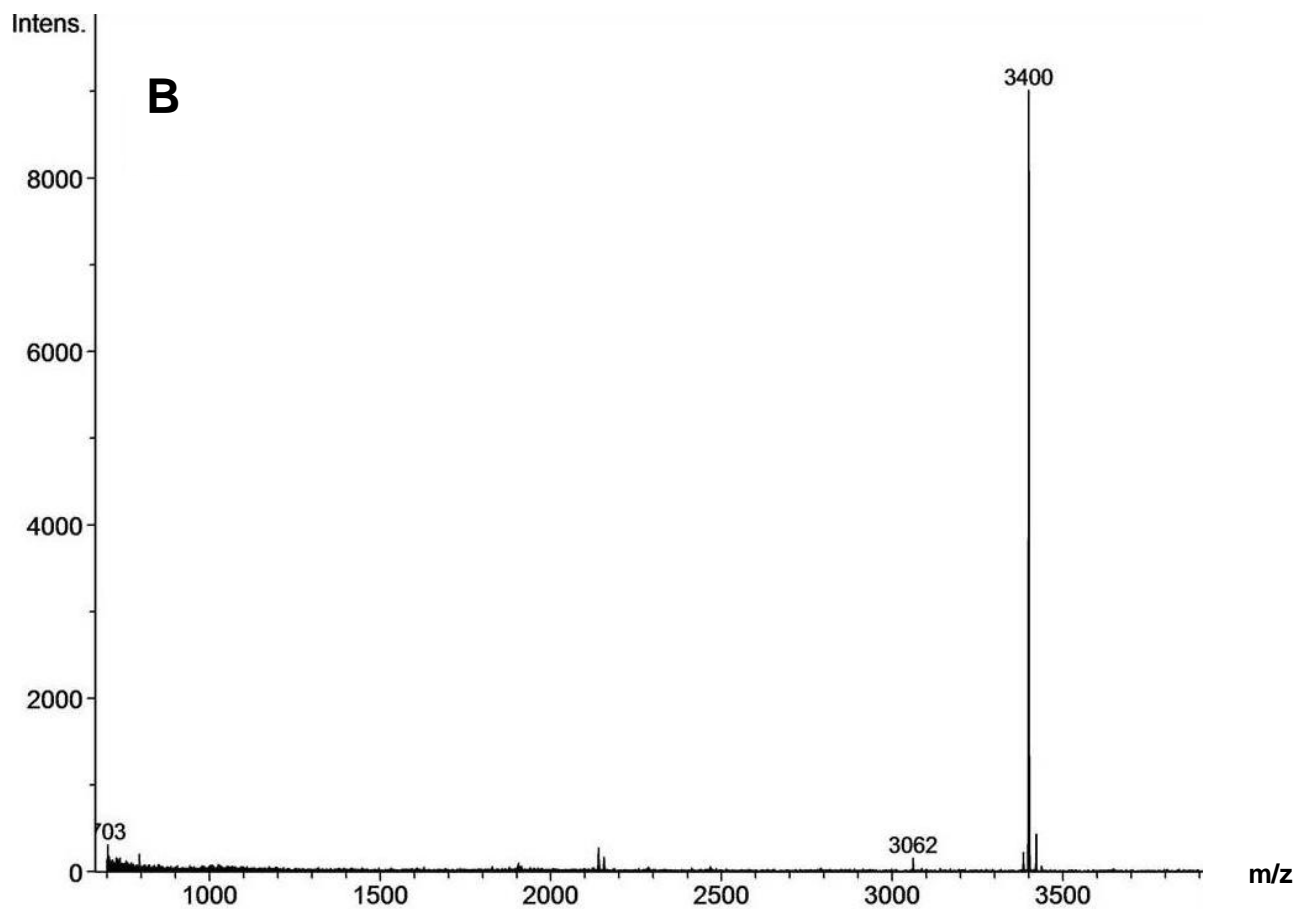

Supplement: S1 Fig — (PDF) [file pone.0140468.s001.pdf]

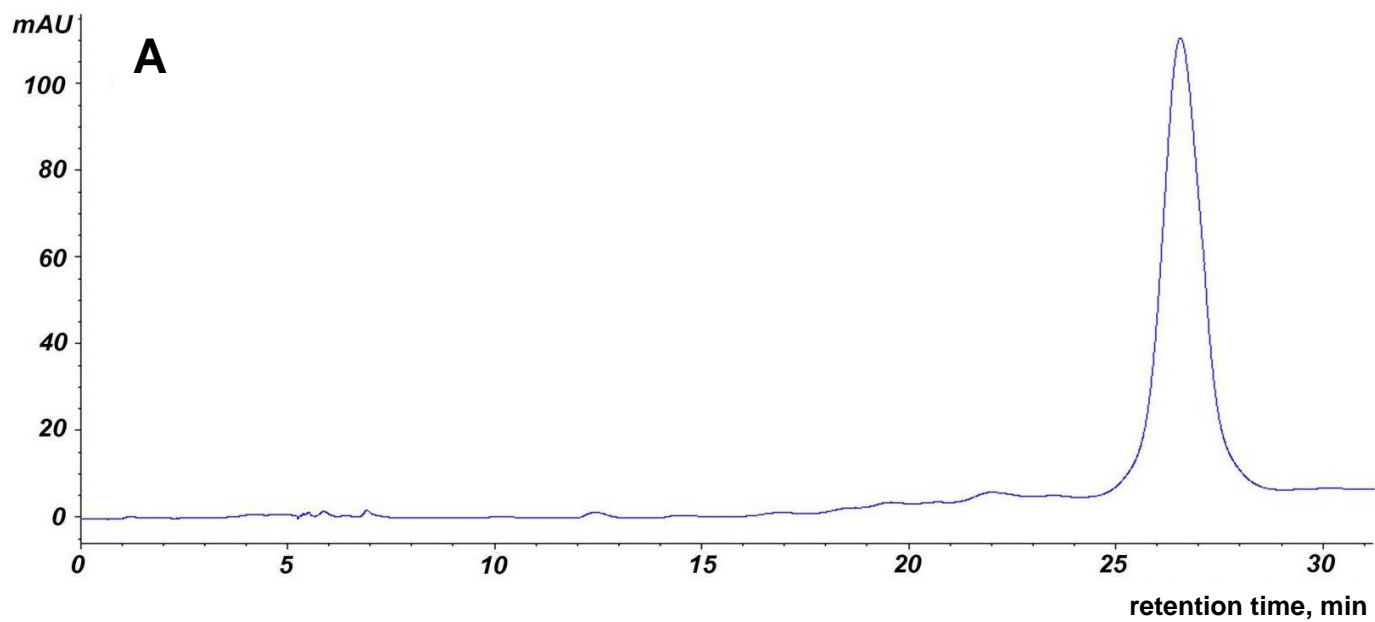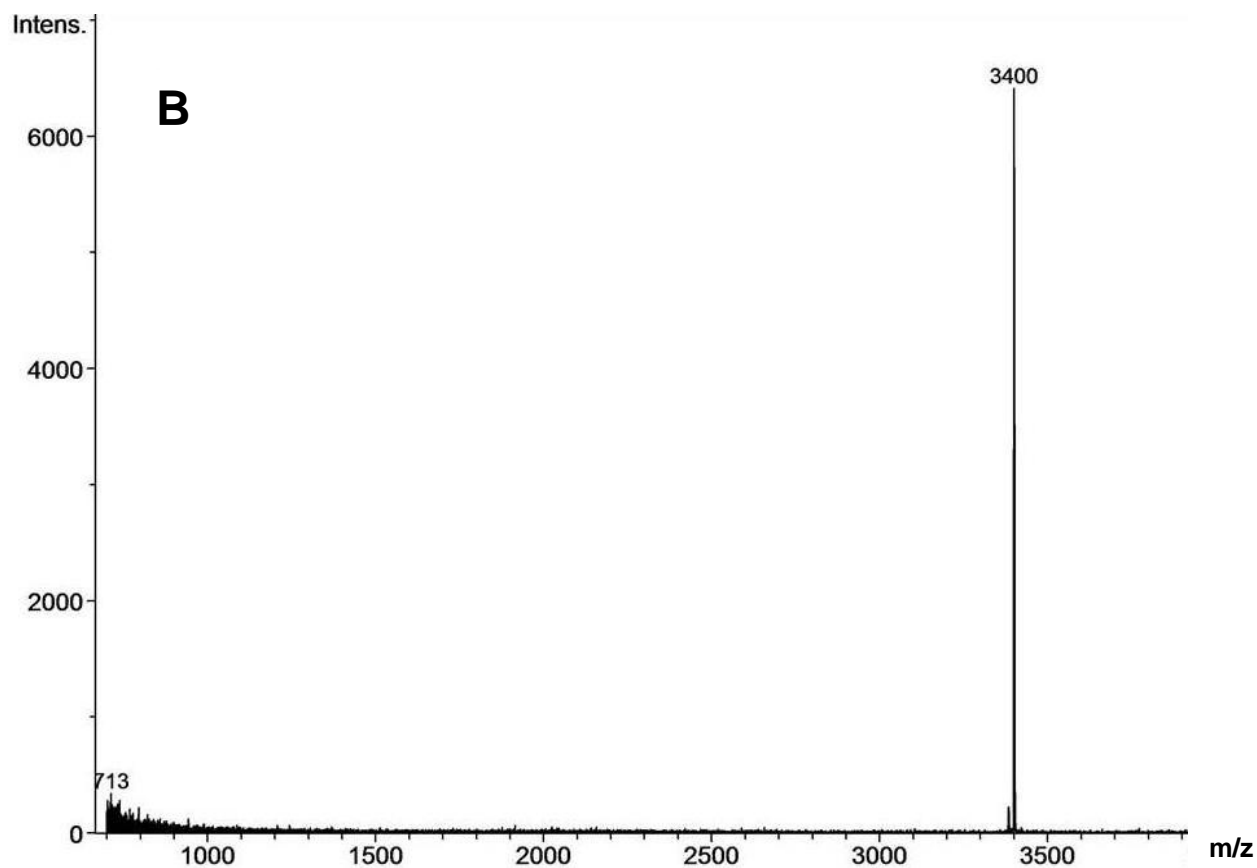

Supplement: S2 Fig — (PDF) [file pone.0140468.s002.pdf]

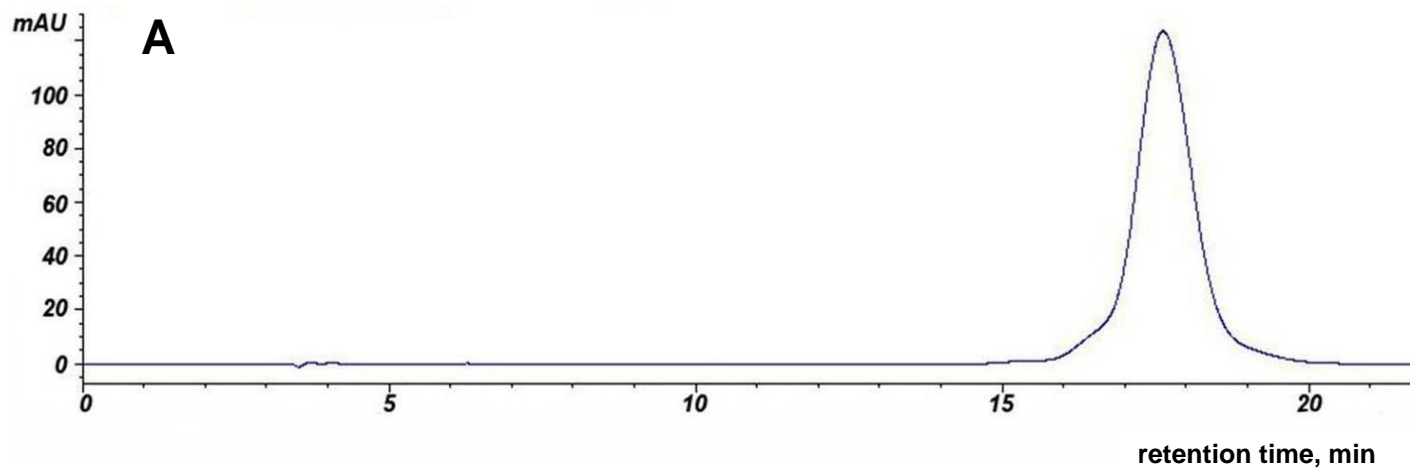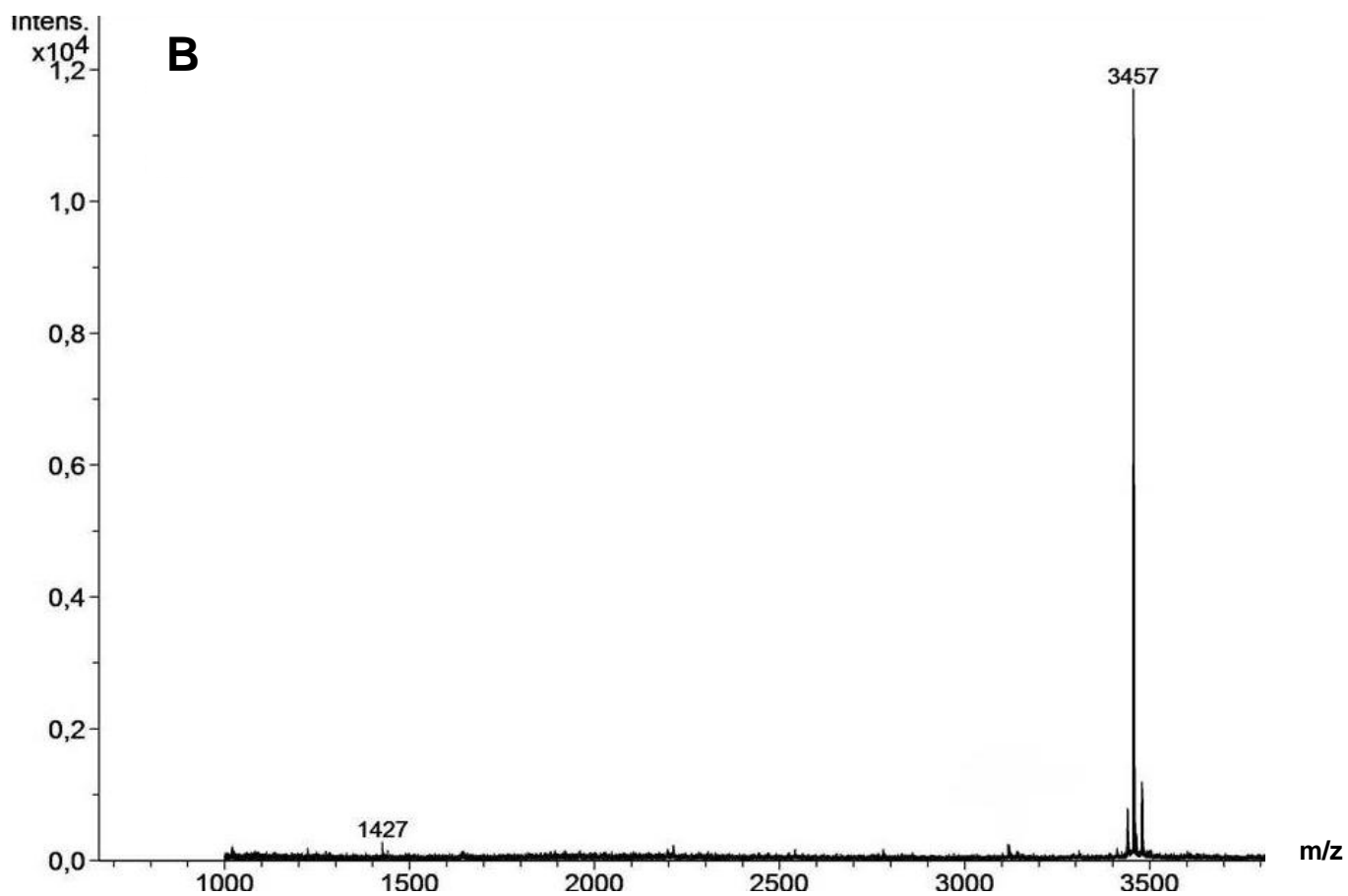

Supplement: S3 Fig — (PDF) [file pone.0140468.s003.pdf]

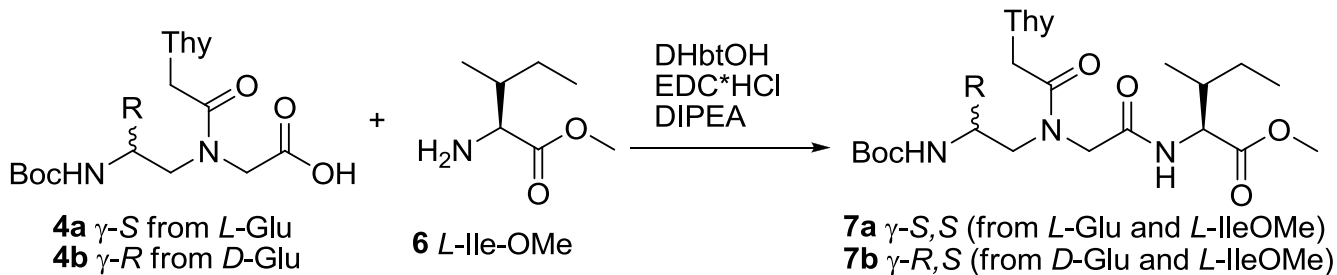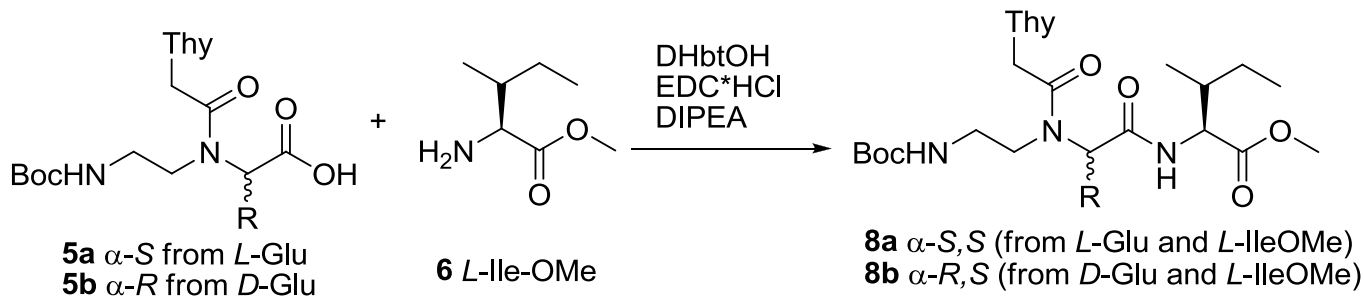

Supplement: S1 Scheme — (PDF) [file pone.0140468.s005.pdf]

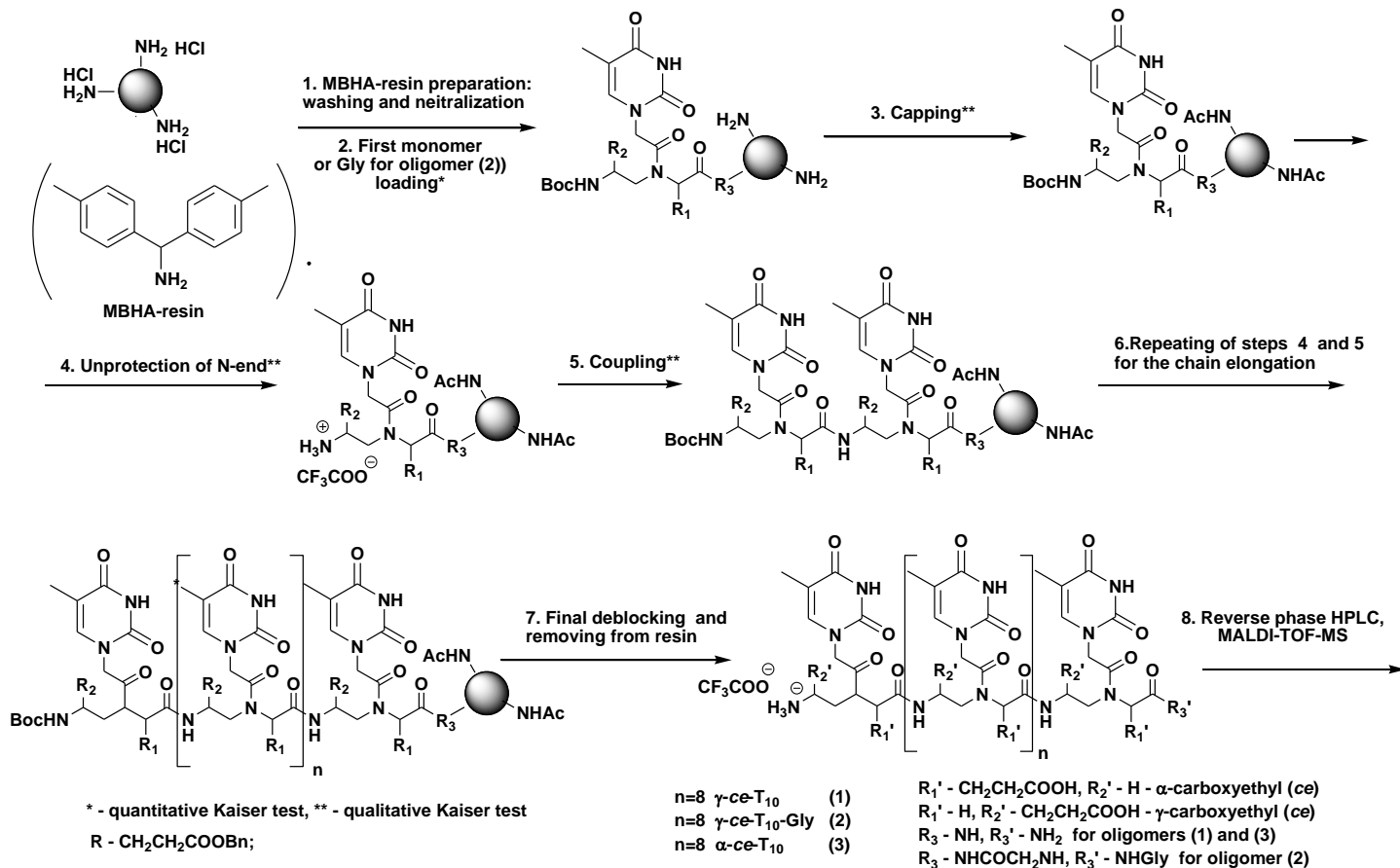

Supplement: S2 Scheme — (PDF) [file pone.0140468.s006.pdf]
