# Supplementary material for: Polyanionic Carboxyethyl Peptide Nucleic Acids (ce-PNAs): Synthesis and DNA Binding
Source: PLoS One. 2015 Oct 15;10(10):e0140468. doi: 10.1371/journal.pone.0140468 (PMC4607454; doi:10.1371/journal.pone.0140468)
Supplement: S4 Fig — (A) Buffer: 10 mM Na2HPO4 (pH 7.4), 140 mM KCl, 5 mM MgCl2. The concentration of each oligonucleotide or PNA was 2.5 μM. (B) Buffer: 10 mM Na2HPO4 (pH 7.4), 10 mM KCl. The concentration of each oligonucleotide was 1 μM; PNA concentration was 2 μM. Tm values were defined as the maxima of the first derivatives of absorption from temperature. (PDF) [file pone.0140468.s004.pdf]

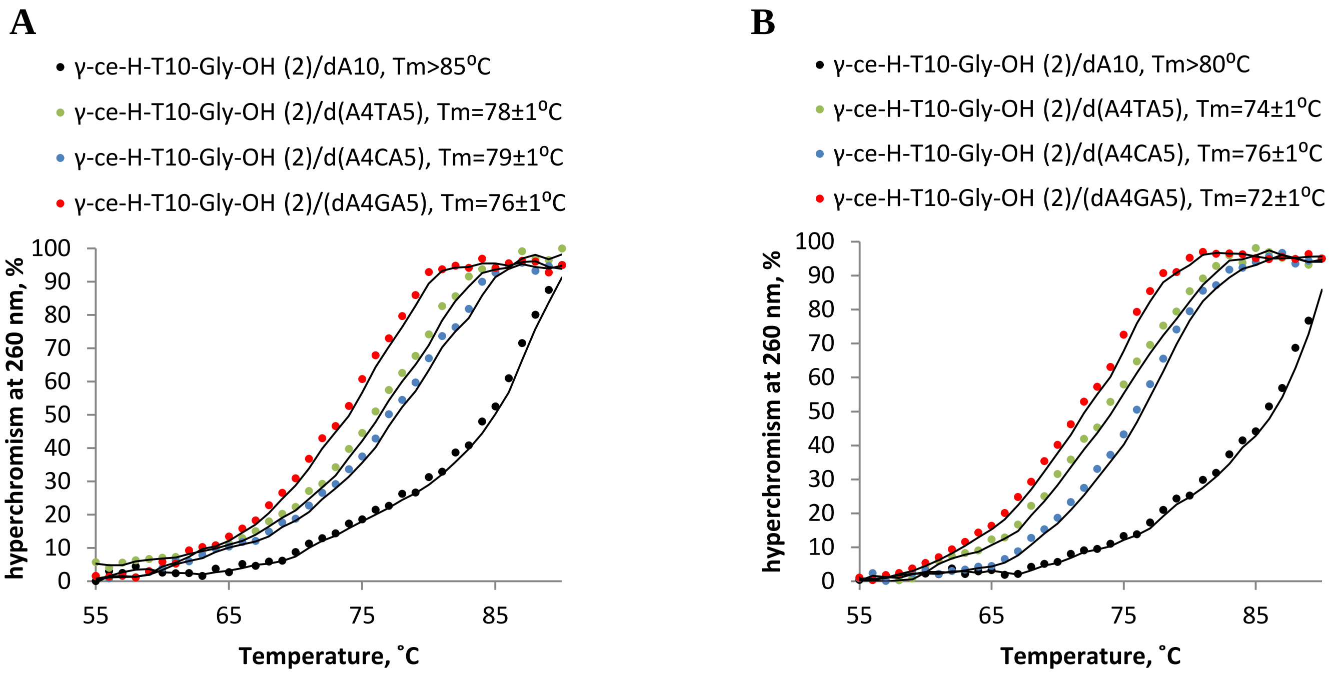
A
γ-ce-H-T10-Gly-OH (2)/dA10, Tm>85°C
γ-ce-H-T10-Gly-OH (2)/d(A4TA5), Tm=78±1°C
γ-ce-H-T10-Gly-OH (2)/d(A4CA5), Tm=79±1°C
γ-ce-H-T10-Gly-OH (2)/(dA4GA5), Tm=76±1°C
hyperchromism at 260 nm, %
Temperature, ˚C
B
γ-ce-H-T10-Gly-OH (2)/dA10, Tm>80°C
γ-ce-H-T10-Gly-OH (2)/d(A4TA5), Tm=74±1°C
γ-ce-H-T10-Gly-OH (2)/d(A4CA5), Tm=76±1°C
γ-ce-H-T10-Gly-OH (2)/(dA4GA5), Tm=72±1°C
hyperchromism at 260 nm, %
Temperature, ˚C
